# Editorial Briefs

**Published:** 1877-10

**Authors:** 


*Editorial Briefs.*—Prof. Taft, (Dental Register,) criticising the amalgam fever, says on the subject : "In nine cases out of ten, when dentists have attempted to write on the desirability and importance of obtaining a plastic material, of universal application, and one that should possess all the qualifications imaginable, they have simply settled down into a fulsome laudation of amalgam, generally, or of some particular form of amalgam, and a condemnation of gold, at least in contrast with amalgam. * * * The burden of the day is amalgam, its efficiency, mode of use, &c. Almost every advocate says it is better than gold, and then he usually squarely contradicts himself before closing. * * During the last thirty years there has been produced upon an average, one new amalgam annually, each of which was better than all others, indeed was about perfect, and so of course could not be superceded. * * The fact that alloys for the most part, and amalgams always, are exceedingly fickle in their behaviour, under varying circumstances has been overlooked, by both those who prepare and those who use amalgam for filling teeth."

He quotes Fletcher as saying, that an "' alloy used for amalgam, whatever its composition, is utterly unreliable unless every ingot is carefully tested for shrinkage, expansion, &c. However good an alloy may be generally, no single sample of it is trustworthy until after a series of tests which cannot be completed in less than three to six months.' And he might have added that even after any given sample is thus tested, where it comes to be combined with mercury, and subject to varying outside influences, such as moisture, heat and electricity, its behavior will lack uniformity.'"

He has seen, he says, many fillings of Fletcher's Amalgam, signal failures. He thinks all amalgams in some mouths oxydize, and in some mouths none will do so.

